# Exsudative Diathesis—A Review

**Published:** 1916-06

**Authors:** Carl G. Leo-Wolf

**Affiliations:** Buffalo, N. Y.


### Exsudative Diathesis—A Review*

By CARL G. LEO-WOLF, M. D., of Buffalo, N. Y.

(Continued from May issue.)

**Symptomatology:** Pfaundler (68) states the case very clearly when he says that some children show a peculiar frequency in the disturbances of their health, no matter how carefully they are guarded against the causes which are known to produce these attacks. This proves, to him, conclusively that these children possess in their organism some peculiarity in the form of a special disposition, an increased readiness towards certain disturbances, a diathesis. Such a diathesis is not, however, a disease, but only a predisposition towards some forms of illness. Different of these dispositions may combine in one and the same individual. As to the manifestations of exsudative diathesis, he differentiates between primary, secondary and concomitant symptoms, as follows:

| | Primary | Secondary | Concomitant to secondary |
|---|---|---|---|
| On skin | Seborrhoea of scalp and face intertrigo prurigo | Eczema, impetigo, abscesses | Scratching, restlessness, easily frightened, disturbances of sleep. |
| On mucous membranes | Conditions of desquamation, swelling of different parts which are evanescent | exsudative processes angina palatina and pharyngea pharyngitis | high fever, cough, vomiting, refusal of food. |
| | | gastro-enteritis | vomiting, pylorospasmus, colic, constipation, muco-membranous diarrhoea, |
| | | coryza | hay-fever |
| | | laryngitis | pseudo-croup |
| | | bronchitis | spasmodic cough |
| | | bronchiolitis | bronchial asthma |
| | | conjunctivitis phlyctena blepharitis | blepharospasm |
| | | balanitis vulvo-vaginitis cystitis | dysuria, ischuria |
| On lymphatic organs | Hyperplasia of palatine and pharyngeal tonsils hyperplasia of palpable glands in neck and around joints. | | |

**Skin:** The external integument being the one organ which naturally will show the changes due to exsudative diathesis most readily and at the same time most clearly, is therefore the organ in which this condition has been studied very closely.

According to Feer (21) eczema is the expression of a chronic constitutional abnormality. There is no acute eczema only acute exacerbations. Acute dermatitis is mistaken for acute eczema, as is proven by the results of treatment. Eczematous conditions are not skin diseases "sui generis," but they are only the symptoms of a constitutional abnormality, often in connection with external stimuli. He prefers the term "eczematisation" which has been coined by Besnier, rather than eczema. The diathesis is the constitutional abnormality, the prevailing disposition of the organism, which leads to eczematisation under the action of contributory external noxes. Scrofular eczema is not merely the sum of tuberculosis and eczema, but through the tubercular infection of the organism the eczema acquires a changed character which gives it its specific appearance.

Bloch (7) sees in the diathesis a chemical allergia. To him the diathetic dermatosis is a reaction of the allergetic skin to accidental irritation, which latter may be either endogenous or exogenous in origin.

Raschmilewitsch (73) found that slight injuries to the skin, followed by irritation of the same (by mustard) caused a typical reaction, not only in children with manifest exsudative diathesis, but also in the latent cases, and in the newborn.

Mautner (56) on the other hand finds no difference between healthy and eczematous children in their reaction to cataplasms or to bacterial irritation, and that their reaction to mechanical and chemical irritation is slight and not specific. He comes to the conclusion that a specific relation between eczema and vasolability, reaction to maceration and to bacterial proteins cannot be proven.

Hirschberg (32) puts stress on the importance of heredity, disposition, disturbances of digestion and metabolism, for the development of eczema, especially in children.

Schlesinger (81) found the weight of infants with universal eczema and still more of those with localized eczema, especially during the first three months of life, not only to be good, but higher than in other diseases of infancy. At the time when the eczema develops an excessive or at least an increased gain in weight may frequently be observed. Only in moderately nourished infants with a very acute development of the eczema did he observe a standstill or a slight loss in weight. Whilst the gain is continuous and excessive the eczema will be very persistent, improvement can only be noticed when the gain is again down to the normal.

Schkarin (80) never missed other symptoms of a constitutional abnormality with eczema, such as exsudations into the skin and mucous membranes, which influence the course of nutrition. The diathesis comprises functional disturbances of the organism and also sometimes hyperplasia or aplasia of some organ of internal secretion. Some eczemata he has been able to cure by simple dietetic measures, whilst in others the treatment is powerless and they will heal by themselves; these latter are the cases which are combined with spasmophilia, in which the exsudative diathesis and the spasmophile diathesis must be treated together. Of the children with eczema up to ten years of age sixty per cent had it during the first two or three years of life.

Moro & Kolb (62) see the inclination to intertrigo in early infancy, to urticaria later, and to reactive inflammations at school-age to be due to a liability in the vasamotors. They frequently observe in the other children of the families of their eczema patients sudden changes in color, headaches, fainting spells, perniones and sudores.

**Lymphatic Apparatus:** Czerny (13) considers the affections in the lymphatic glands in exsudative diathesis never to be primary, but always to be secondary to pathological processes in the skin and mucous membranes. The condition of the lymphoid organs, the thymus, spleen, tonsils, intestinal follicles is independent of that of the lymph-glands. Their hypertrophy is due to overfeeding which favors the depositing of fat.

Benfey & Bahrdt (5) studied thirteen cases, none of whom had infantile eczema nor tuberculosis; these children were taller and lighter than normal, their skin was tender and transparent, they looked pale but not anemic, they had long eyelashes and long silky hair. The clinical picture in these cases was dominated by adenoids and by regional glandular swellings. The authors sometimes found a slight swelling of other superficial glands, due to frequent infections based upon the diathesis, which latter offers an increased disposition towards diseases. Only individuals thus predisposed react with catarrhs to noxes which do not produce nasopharyngeal troubles in other children. They believe in the existence of a "circulus vitiosus," namely: disposition AND infection, but not disposition OR infection.

**Blood:** Kroll-Lifschütz (43) regards eosinophilia as a coordinated symptom of exsudative diathesis, he found the following counts:

In healthy children .............Eosinophiles 1.8 to 2.8%<br>
In eczema children ................Eosinophiles 5.5 to 26%<br>
In children with strophulus or asthma Eosinophiles increased<br>
In children with lingua geographica<br>
or recurrent catarrhs.............Eosinophiles normal.

Helmholtz (31) and Rosenstern (76) both find eosinophilia accompanying eczema.

Putzig (70) has investigated a large material with the most careful methods. He finds that healthy children have the same eosinophile count as have adults, namely 2 to 4% whilst infants who later show signs of the exsudative diathesis have an increase in the eosinophiles quite early, especially in the third or fourth week of life. Acute infections and digestive disturbances cause a diminution of the eosinophiles. Typical eosinophilia was demonstrated by him in infants with skin-affections due to primary exsudative symptoms, but not with universal infected eczema nor intertrigo. Nor did he find eosinophilia to conform to the severity of the skin-eruption, as it outlasts this latter. He concludes that eosinophilia is a symptom of exsudative diathesis which may be regarded as a type of a protracted anaphylaxis.

Aschenheim (2) on the other hand considers eosinophilia as a special disposition, an eosinophile diathesis, which is not identical with exsudative diathesis but frequently combined with it. He comes to the following conclusions (1):

1. Eosinophilia is more frequent with florid eczema than with other manifestations of exsudative diathesis;
2. Eosinophilia disappears with the improvement of the skin-affections;
3. Eosinophilia cannot be regarded as a valuable symptom of exsudative diathesis;
4. Eosinophilia has not been found as a family-peculiarity where several cases have been observed in one family;
5. Eosinophilia may be found in healthy people who have never shown any signs of exsudative diathesis.

**Respiratory Apparatus**: Czerny (12) sees a great similarity in the affections of the air-passages to those of the skin. His views differ greatly from those of the French school, who consider asthma and bronchitis to be due to arthritism or herpetism, as for instance Goilav (25), Merkel (59) and Spolverini (87). Some children suffer from repeated affections of the same parts of the respiratory mucous membrane and in the same manner. One child has pharyngitis several times a year, another follicular angina, a third an infection of the pharyngeal tonsil, a fourth pseudo-croup, a fifth bronchitis diffusa. The different attacks are very similar, and we must therefore assume that the same local disposition exists as in seborrhoea, the difference consisting only in the form of the reaction to an equivalent pathological irritation.

Multiple infections of the air-passages must be regarded in the same manner, the irritation giving the chance for the infection, the same as it does in the skin. These children may frequently escape infection as long as they live in the country, but when they move into the city, they will acquire infections and their general health will then suffer. This explains inversely the benefit these children derive from climatic changes, which, however, do not cure the exsudative diathesis, because upon the return of the child to its former surroundings the infections will reappear.

The irritation in the upper air-passages will cause an increase in the lymphoid tissue of the tonsils even in children who have not suffered from infections, though this will be more pronounced after repeated infections. The view that the enlarged tonsils cause the disposition to the infection of the naso-pharynx is erroneous, and this is proven by the fact that the infections continue after the removal of the tonsils. The apparent beneficial effect of the removal of the tonsils around the tenth to twelfth year is due to the fact that at this age the exsudative diathesis is on the wane. The infections do not start in the tonsils but from the mucous membrane around these.

The pathological conditions in the mucous membrane of the upper respiratory tract cause, in cases of exsudative diathesis, loss of appetite, which however is not a continuous anorexia, but one which comes on for longer or shorter periods, following periods of normal or even excessive appetite. During the anorexia the tongue is coated. These children have foetor ex ore from the disintegration of exsudates in the crypts of the adenoids. Sometimes they have constipation, and perhaps fever for a few days, but rarely longer than a week. Some children have malaise at this time, while others feel well though they are pale, this latter depends upon the irritability of the nervous system only. The children with large pharyngeal tonsils are frequently treated with strict diet for gastric disturbances, a so-called spoiled stomach.

When the exsudative diathesis appears in other parts of the respiratory mucous membrane the general condition of the child is frequently overlooked, especially is this the case in follicular angina. Some children with pharyngitis cough considerably, others do not, this depends upon the reaction of the nervous system.

We will also observe an interchangeability of the symptoms in the same child, for instance between follicular angina, pseudo-croup and asthma.

The so-called asthma of children, which was first described by the French school, is nothing else but an acute diffuse bronchitis, and it is observed very frequently in exsudative lungs, which may be audible at a distance, together with children. In these we hear first a whistling noise over the accelerated and shortened respirations; then we hear râles, which are more or less confined to the large bronchi, whilst the asthmatic symptoms depend upon the nervous condition of the child, in the same manner as the itching in prurigo.

Some children have these attacks of bronchitis every few weeks or months, but without asthmatic symptoms, whilst others will become more and more asthmatic. We must, however, remember that the diffuse bronchitis is a part of the exsudative diathesis and not the asthma.

**Digestive Apparatus**: Lingua geographica—also called wandering rash, Landkartenzunge, état lichénoid, desquamation epithéliale, benign plaques, lingua maculata—is according to Groos (28) not a parasitic invasion but most likely constitutional, and is found in the hereditary-neuropathic type of individuals. He believes that a pathologic-anatomical basis for it can surely be found.

Lublinski (51) denies its connection with exsudative diathesis.

Klausner (39) considers it a congenital debility and consequent irritability of the mucosa of the tongue, and he states that dermatologists do not agree that it is part of the exsudative diathesis, and that they are still looking for its etiology.

Czerny (12) however, regards it as one of the first symptoms of exsudative diathesis, which may at times be seen during the first month of life, but only in the living child. According to him it is a painless exsudation into the mucous membrane which makes the papillae appear enlarged and prominent. The increased desquamation makes the affected parts to look white. It is very evanescent and not depending upon the general health of the child.

Lieblein (50) examined six cases in young people with pronounced lymphatism, but in no case did he find, either macroscopically or microscopically, an inflammation of the appendix; he found, however, a considerable hyperplasia of its lymphatic apparatus and very long appendices. Although the symptoms disappeared in every one of his cases after operation, he has not yet been able to find a satisfactory explanation for this condition.

Langstein (46) could not find any pathogenic organisms in many cases of infants in whose stools he had noticed pus. These infants had the clinical manifestations of exsudative diathesis, and he believes that in exsudative diathesis a special disposition to intestinal symptoms exists, which will result in mucus in the stools in light cases, and pus in severe ones. In two of his cases he also found eosinophilia of a pronounced type. He sees in the hyperplasia of the intestinal follicles, which was first described by Czerny (13), the reason why infants with exsudative diathesis react to noxes which do not affect healthy infants.

**Genito-urinary Apparatus**: Lust (52) found in the sediment of apparently normal urine, which he had centrifuged, epithelium and a few leucocytes as a sign of a process of desquamation in the uro-genital mucosa in more than fifty per cent of the children with exsudative diathesis. He sees in this a possible explanation why pyelo-cystitis in boys is more frequent with exsudative diathesis.

Beck (3) in his study of forty cases of his own confirms Lust's findings of desquamative processes on the mucosa of the efferent urinary passages.

**Nervous System:** Saenger (78) considers the psychic element very important as supplementing the somatic disposition of exsudative diathesis.

Czerny (14) lays stress upon the close connections between the neuro-psychopathies and exsudative diathesis, though he does not look at this as causative but only as a combination. Frequently the education of the child has been erroneous, owing to the exsudative diathesis and thus the neuro-psychopathies are favored. This explains the remarkable results which have often been observed under psychic treatment in children with obstreperous eczema or asthma, sometimes also in processes originating in the naso-pharynx. Fattening or rest-cures or medication do not help here, only radical changes in the mode of living and in the education of these children will be effective, and this often only if carried out away from home.

Pfaundler (66) calls the attention of the physician to the cases, which we see so frequently, in which the symptoms of the lymphatic constitution are combined with those of neuropathy, to form, what he calls neuro-lymphatism. In these cases the nervous manifestations, the severity of the reflexes. are of special importance, as for instance the spasmodic sneezing in coryza, the pseudo-croup in laryngitis, the pertussoid in bronchitis, the asthma in bronchiolitis, severe colic and muco-membranous diarrhoea in enteritis, enuresis in balanitis, blepharospasm in conjunctivitis. Pediatrists know that these symptoms are usually observed in children who had been overfed with milk and eggs, and whose education had been faulty, whilst race and nationality are of no importance. The treatment of these patients is psychic and dietetic, suggestion with severe shocks is often beneficial.

Czerny (12) explains that the severity of the itching is dependent upon the degree of irritability of the nervous system. The itching causes the children to scratch, and then the danger of a secondary infection and thus an eczema is great. He also states that the less the morbidity of the child, the better will be its physical and psychical development. He warns physicians not to neglect the child's psyche over its body. He also warns against the dangers of making the child neuropathic by letting it feel that it is always a patient. Polypragmasy must, therefore, be avoided, the attention of the child must be detracted from its own body, nobody must talk about the child's looks or bodily functions in its presence. The child with exsudative diathesis must not be made to feel that it is sick or that it requires special care. It will best be as much as possible in the company of other children of the same age, and as little as possible with adults. The external signs which will warn us that we should pay more attention to the child's psyche, are frequent and sudden changes in its color, which are not due to anemic but to vasomotor disturbances. The avoidance of accidental affections will also have a beneficial effect upon the child's psyche, because sickness interferes with its education and favors neuropathy.

**The Eyes**: Igersheim (36) found in seventy per cent out of 152 children with phlyctena a positive v. Pirquet reaction. He therefore looks upon patients with phlyctena as tuberculous, or at least highly disposed towards tubercular infection. He urges in these cases the treatment of the exsudative diathesis, and he advises great care in keeping these children away from people suffering from tuberculosis.

**Metabolism**: Menschikoff (58) has investigated the metabolism of children with exsudative diathesis and he finds that they react easier to differences in the ingestion of chlorine, increases as well as decreases, and more so with manifest than with latent exsudative diathesis.

Bernis (6) and Kern (38) look upon the change in the nitrogen-metabolism as characteristic. The latter found that children with exsudative diathesis have a delayed excretion of uric acid, and he assumes from this a connection to exist between this diathesis and arthritis urica.

Lederer (47) blames a disturbance in the metabolism of water.

Steinitz & Weigert (89) find a decrease in the metabolism of fat and nitrogen.

Czerny (15) is convinced that the metabolism of fat is disturbed.

Finkelstein and Meyer claim to have found the reason for the changes in the altered metabolism of the salts.

**Connection with other Diseases**: v. Hansemann (29) states that many diseases develop upon the basis of either acquired or congenital constitutional abnormalities, which consist in anatomical or metabolic changes. The composition of the body-juices and the activity of the cells form the medium through which the growth of the pathological elements may be either favored or destroyed. Some causes, like colds, or general diseases, like gout or ricketts, also age and inheritance will cause a change in the composition of the body-juices.

Unterberger (93) sees in the lymphatic constitution a factor which is of paramount importance, especially in the development of tuberculosis, against which the action of the bacteria may be neglected. In the prophylaxis of tuberculosis this constitutional abnormality must be corrected first of all and the destruction of the bacilli is then of minor importance.

Rozenblat (77) is doubtful about the connection between scrophulosis or tuberculosis, which he regards as identical, and exsudative diathesis.

Czerny (17) clearly defines his standpoint when he writes: The identical infectious agent does not produce similar clinical pictures in different individuals. This is due only partially to the quantity or quality of the microorganism or its location in the body. The appearance of certain symptoms of infectious diseases and their course depends mainly upon individual constitutional abnormalities. These may be either congenital or acquired, they may affect either single organs or the whole body.

Thus only a very small number of infants die solely from uncomplicated digestive disorders. The majority of these infants get sick owing to their abnormal disposition whilst they are taking a food which would not seriously damage normal infants.

The same is true of the infectious diseases in older children, in whom the influence of the neuropathic constitution is especially strong, due to the lability of the innervation of the blood vessels. This is very pronounced in pertussis.

The normal human tissue possesses an excellent mechanism for the regulation of the retention of water, in some, however, this is congenitally defective, they will then have a tendency towards a considerable retention of water, which may even lead to edemata, and will also cause a great lability in the body-weight. A pathological retention of water is seen most frequently under a carbo-hydrate diet, and these children are then often considered plump. As infections are very dangerous to water-logged children, we must reduce the water in the body prophylactically, and this especially as regards tuberculosis, since we know that miliary tuberculosis and tubercular meningitis occur mostly in stout children. Czerny warns for the same reason against the overfeeding of tuberculous children.

Engel (19) is of the opinion that scrophulosis develops upon the soil of exsudative diathesis, the infection with the tubercle bacillus being added to the constitutional abnormality. Tuberculosis can also increase or bring out the symptoms of exsudative diathesis, the same as would any other infectious disease, the more so because tuberculosis causes a long-continued irritation, which makes the symptoms of the exsudative diathesis so much severer. On the other hand tuberculosis may also cause in children with exsudative diathesis a severe allergia as shown in the much increased sensitiveness to tuberculin; this is what makes scrophular symptoms so characteristic.

If we add to this, amongst the poorer classes, poor food, dirt and deficient hygiene, we will get so much more readily the inflammations and catarrhs which are based upon the constitutional sensibility and which are increased by the sensibility due to the infection. This then will produce the habitus scrophulosus.

Scrophulosis must therefore be regarded as a disease produced by the cooperation of exsudative diathesis and tuberculosis, though other noxes may produce similar clinical pictures in cases of exsudative diathesis.

(To be continued in July issue.)

---

**Anthrax Due to Shaving Brush.** The Hospital mentions the case of a man who died in the West London Hospital last summer after an illness of 2½ days of cellulitis of the neck determined post mortem to be anthrax. He had just begun using a new shaving brush which, fortunately, had not been washed subsequently. Anthrax bacilli were found in this brush and also in 4 out of 5 new brushes of the same batch. (Note: While rinsing with hot tap water at approximately 70 degrees C. kills, inhibits or mechanically removes most germs, bacteria or others, liable to be present or to be conveyed to brushes, it is a wise plan to soak for an hour or so a new tooth, nail or shaving brush in strong bichlorid solution after preliminary washing and before putting the brush into commission).

---

**Paravaccinal Typhoid Hepatitis.** Sirot, La Caducee, Dec. 15, 1915, calls attention to the fact that after typhoid vaccination, there may be a more or less delayed malady, resembling typhoid, even to the appearance of rose spots and phlebitis. This occurs in two distinct types, one a general condition, the other localized in the liver which he considers a hepatitis.

---

**Early Diagnosis of Eruptive Fevers by Use of Cupping Glass.** d'Oelsnitz, Soc. Med. des Hop., Oct. 22, 1915, advocates this method to develop the rash early. Measles shows itself as a persistent image like a geographic chart. Scarlatina shows a fine, irregular ecchymotic stippling. If a rash develops ecchymotic patches, it is a prognostic indication of a haemorrhagic tendency.

